# Effect of freezing-thawing on the quality changes of large yellow croaker treated by low-salt soaking during frozen storage

**DOI:** 10.3389/fnut.2022.1103838

**Published:** 2023-01-10

**Authors:** Hongli Bao, Jinsen Zhang, Mingao Li, Yi Chen, Chunyan Mao, Jing Yang, Yuanpei Gao, Shanggui Deng

**Affiliations:** ^1^Key Laboratory of Health Risk Factors for Seafood of Zhejiang Province, College of Food Science and Pharmacy, Zhejiang Ocean University, Zhoushan, China; ^2^School of China Alcoholic Drinks, Luzhou Vocational and Technical College, Luzhou, China

**Keywords:** large yellow croaker, freeze-thaw cycles, frozen storage, salting, microstructure

## Abstract

**Introduction:**

The production of the large yellow croaker has seasonal and regional characteristics, which is typically preserved on ice, possibly leading to its deterioration in a short time. Therefore, in this study, we focused on the effect of temperature fluctuation on the quality changes of the large yellow croaker during frozen storage.

**Methods:**

In this experiment, the large yellow croaker was soaked in a low-salt solution, and physical and chemical properties, water-holding capacity, color, and protein characteristics of the muscle were investigated after repeated freeze–thaw (F–T) cycles and frozen storage.

**Results and discussion:**

The results show the deterioration of muscle quality of large yellow croaker after low-salt treatment was lower than that of the salt-free soaking group. The salting treatment significantly (*P* < 0.05) enhanced the yield of large yellow croaker, which was 24.3% greater than the salt-free soaking group after 6 weeks of frozen storage. The microstructure of the salted muscle was more stable and maintained its cellular structure after F–T cycles and frozen storage. The b* value of the salt-free soaking group increased from b* value of the low-salt soaking group decreased from acceptable range. Sodium dodecyl sulfate polyacrylamide gel electrophoresis (SDS-PAGE) analysis indicates the content of 17 kDa peptide decreased in the low-salt soaking group, and the peptides at 21 and 24 kDa increased during frozen storage. The results of the present study provide guidance for the optimal processing, transport, and storage of large yellow croaker, but the effect of salting on lipid oxidation and protein oxidation requires further study.

## 1. Introduction

According to the Food and Agriculture Organization of the United Nations (FAO), the annual production of artificially farmed large yellow croaker (*Larimichthys crocea*) (LYC) reached 254,000 tons in 2020 (1). Currently, the primary preservation model is ice temperature storage; however, storage is limited to a maximum of 8 days, due to the high protein and fat content (2). Freezing technology extends the shelf life of LYC by inhibiting microbial and enzymatic activity. Aquatic products have regional and seasonal characteristics, and frozen storage can effectively extend the shelf-life of aquatic products, thus providing convenience for consumers anytime and anywhere. However, the deterioration of muscle during frozen storage leads to decreases in water holding capacity and increases the thawing loss. As a result, a decrease in product quality is observed (3). In addition, cold chain logistics during transportation are not able to link to every single section, resulting in large fluctuations in the temperature of frozen food. Therefore, repeated F–T cycles occur, which also leads to a decrease in product quality (4, 5). Repeated F–T cycles result in increased accumulation of ice crystals in food, and recrystallization exacerbates mechanical damage to muscle tissue (6). Repeated F–T cycles affect the protein integrity and increase the rate of thawing loss (7). F–T cycles have become a focal point in the transportation of aquatic products.

Salting, as a traditional method to preserve food, can inhibit the growth of microorganisms and active enzymes (8). However, high salt intake leads to cardiovascular and cerebrovascular diseases (9). In recent years, an increasing number of researchers have focused on low-salted foods, which have an extended shelf life, as well as provided unique flavor, texture, and color of food (10). Low-salt shrimp paste showed an umami taste similar to raw shrimp in the study of Yu et al. (11). The low-salt treatment enhanced the water-holding capacity of tuna muscle after repeated F–T cycles in the study of Jiang et al. (12). Salt preservation is commonly used for livestock meat, but salting is rarely used to preserve aquatic products. To explore the application of low-salt preservation for aquatic products, the changes in the quality of LYC, treated with low salt, were investigated during F–T cycles and frozen storage.

## 2. Materials and methods

### 2.1. Sample preparation

Fresh LYC was purchased from the Zhoushan Aquatic market. The samples with uniform size (Weight of 554.8 ± 21.2 g and length of 24.3 ± 2.1 cm) were selected, packed in a polyethylene foam box, and brought back to the laboratory within 30 min under chilled conditions. The fresh Large yellow croaker was separated into the back and abdominal muscle after removing viscera, head, bones, and skin at 4 ± 0.5°C. The muscle was washed in cold deionized water, and the surface moisture was removed with a paper towel. The back muscle was cut into fillet with length of 60 mm before salting. The low-salt soaking (LSS) group was soaked in a 5% sodium chloride solution and stirred continuously. The salt-free soaking (SFS) group was soaked in deionized water. The ratio of material to liquid was 1:4 (w/v), and the salting temperature was controlled at 2–8°C. The fillets were taken out after 4 h. The surface moisture was removed, and the muscle was vacuum-packed using nylon and polyethylene bags. The vacuum-packed fish fillets were frozen at –18 in the refrigerator and subjected to 0, 2, and 4 F–T cycles (each cycle was performed at –18°C for 24 h, followed by 4°C for 12 h). Then, the samples were frozen at –18°C for 6 weeks. The histology, quality, and physicochemical properties were measured every two weeks.

### 2.2. Yield, water content, thawing loss, centrifuging loss

Thawing losses and centrifugal losses (1,760 × *g* for 10 min at 4°C) were determined according to the method of Du et al. (7). The yield was determined according to the method of Jiang et al. (13), and water content was determined via the AOAC method (14) to express the change in the quality of the LYC. The following equations were used to calculate the yield, thawing loss, and centrifuging loss.


(1) $$Yield=\frac{W_{2}}{W_{0}}\times 100\%$$

(2) $$Thawing\,loss=\frac{W_{2}-W_{1}}{W_{2}}\times 100\%$$

(3) $$Centrifuging\,loss=\frac{W_{3}-W_{2}}{W_{2}}\times 100\%$$

Where *W*_0_, *W*_1_, *W*_2_, and *W*_3_ were the masses of LYC samples before salting, after salting, after thawing, and after centrifugation, respectively.

### 2.3. Textural profile analysis

Textural profile analysis (TPA) (iTexture, Zhejiang Zheke Instrument Equipment Co., Ltd., Hangzhou, China) of muscle was determined by a texture analyzer according to the method of Jiranuntakul et al. (15), with slight modifications. Briefly, the muscle was cut into 20 mm × 20 mm × 10 mm sections, and then a P50 probe was used with a 30% type variable, with a detection speed of 5 mm/s, a starting minimum force of 0.6 N, and a return distance of 20 mm. Hardness (N), cohesiveness, springiness, and chewiness were obtained as the average of five repeated measurements (3).

### 2.4. Thiobarbituric acid reactive substances

Thiobarbituric acid reactive substances (TBARS) were measured according to the method of GB-5009.181 (16). Precisely, 10 g of minced muscle was added to 50 mL of 7.5% trichloroacetic acid (containing 0.1% EDTA) and shaken for 30 min at 50°C. After cooling to room temperature 5 ml of the filtrate was collected after filtration through a double layer of filter paper, and 5 ml of 0.02 mol/L 2-thiobarbituric acid solution was added. The mixture was reacted in a water bath at 90°C for 30 min and then cooled to room temperature. The absorbance was recorded at 532 nm. A standard curve was prepared using 1,1,3,3-tetraethoxypropane. The malondialdehyde content was expressed as (mg MDA/kg).

### 2.5. Total volatile basic nitrogen

Total volatile basic nitrogen (TVB-N) was measured according to the method of GB-4789.2 (17). Subsequently, 10 g of the sample was weighed and chopped. Further, 75 ml of distilled water was added to the sample and extracted for 30 min. Then, 1 g of MgO was added, and the sample was immediately loaded onto the semi-automatic Kjeldahl nitrogen analyzer (Kjeltec 8400; Foss, Denmark). The total volatile basic nitrogen was determined by distillation. The distillate was collected in a flask containing 2% aqueous boric acid solution and a mixing indicator (methyl red and bromocresol green ethanol solution). Finally, the boric acid solution was titrated with 0.1 mol of hydrochloric acid.

### 2.6. Color

After thawing, the color was measured by a colorimeter (CS-210, Hangzhou Chnspec Technology Co., Ltd., Hangzhou, China). The luminance (L*), redness (a*), and yellowness (b*) of the muscle were recorded.

### 2.7. Observation of tissue microstructure

The muscle was embedded in a 70% ethanol solution for fixation. The fixed samples were dehydrated, embedded in paraffin, stained with hematoxylin and eosin for 5 min each, and cut into 5 mm slices. The paraffin sections were mounted on a glass slide and observed after HE staining.

### 2.8. SDS-PAGE patterns of protein

Sodium dodecyl sulfate polyacrylamide gel electrophoresis (SDS-PAGE) analysis was used to determine the protein patterns of LYC according to the method of Cao et al. (18), with slight modifications. 3 g of minced muscle was weighed, and 27 ml of 5% SDS solution (85°C) was added. The mixture was homogenized for 2 min at 11,000 rpm and transferred to a water bath at 85°C for 1 h. The samples were centrifuged at 12,000 ×*g* for 20 min (XIR centrifuge; Themo, Germany), and 100 μl supernatant was collected and diluted 10-fold. The supernatant was mixed 1:1 with the sample buffer, boiled for 4 min, and cooled to room temperature. Next, 15 μl sample was loaded onto the polyacrylamide gel (12% separation gel, 5% concentrated gel). Electrophoresis of the upper concentrated gel was performed at a constant current of 40 V, and the constant current for the separating gel was 120 V. After staining and decolorization at the end of electrophoresis, a gel imaging analysis system was used for analysis.

### 2.9. Statistical analysis

Statistical analyses were performed using the SPSS software package (SPSS 17, SPSS Inc., Chicago, IL, USA), and Duncan’s test was used for significance analysis of within-group differences, where the mean was significant at *P* < 0.05. Data are expressed as the mean ± standard deviation (SD).

## 3. Results and discussion

### 3.1. Yield

**FIGURE 1:**
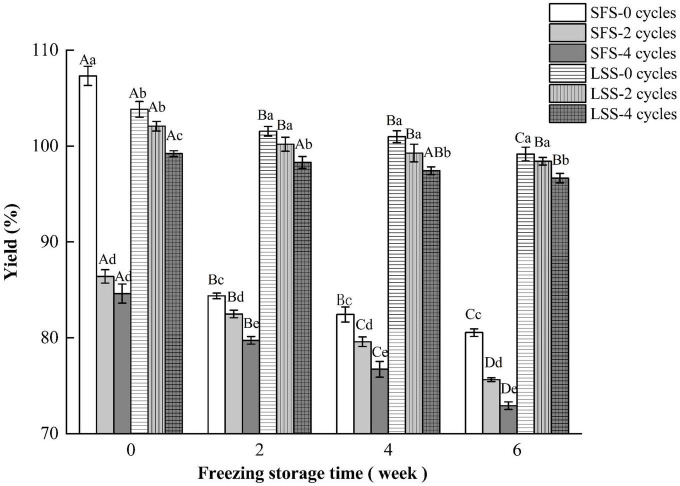
Variation in yield of LYC during frozen storage. SFS and LSS represent the salt-free soaking group and low-salt soaking group, respectively. Lowercase letters represent significant differences between six samples during freezing, and capital letters represent significant differences between samples of different treatment groups during freezing (*P* < 0.05).

The yield of the product is a very important economical factor, and the changes in the yield of different samples are shown in Figure 1. The quality of the LYC increased after low salting, which is also reported by Jiang et al. (19). Fish soaked in salt solutions at concentrations below 15% gained water and salt from the solution. When the concentration of salt increased to 20%, the water mass of the fish muscle decreased due to osmotic pressure (20). Studies have shown that salting increases the yield of fish meat after repeated F–T cycles and during frozen storage (12). The yield of SFS group significantly decreased with increasing F–T cycles and storage periods. The SFS group showed a significant decrease in yield at 0 w, after two and four F–T cycles, and this was due to the increased rate of thawing loss after freezing. The yield of the LSS group after freezing was significantly higher than that of the SFS group, and the yield of the LSS group was 24.3% higher than the SFS group after four F–T cycles and 6 w of frozen storage. The increase in yield was attributed to the increased salt and moisture in the meat. In addition, the increase in water holding capacity had a significant effect on yield. The myofibrillar meshwork of myogenic fibrillar proteins could combine the water in presence of salt, therefore, swelling of myogenic fibrillar leading to an increase of weight.

### 3.2. Texture

**FIGURE 2:**
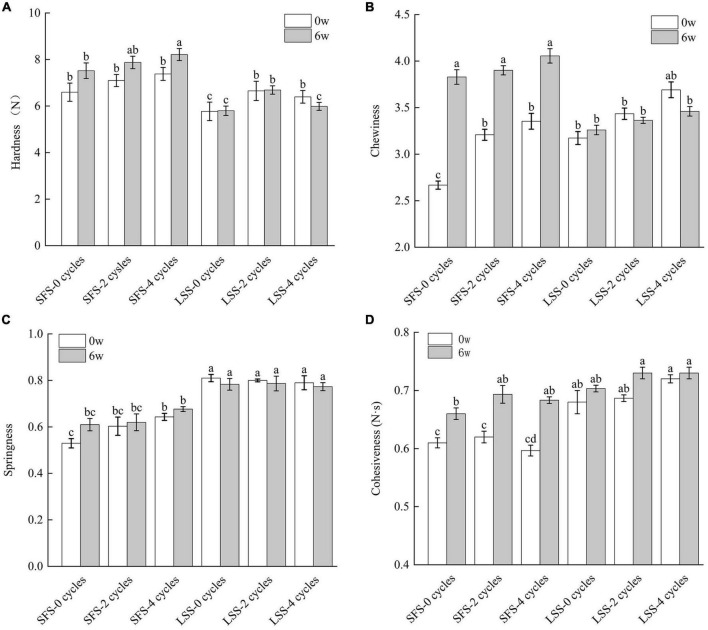
Texture changes of LYC during frozen storage; panels **(A–D)** present the hardness, cohesiveness, springiness, and chewiness, respectively. SFS and LSS represent the salt-free soaking group and low-salt soaking group, respectively. Different letters in the figure represent significant differences between the six samples (*P* < 0.05).

The texture is an important quality indicator of food, and different texture parameters are shown in Figure 2. The hardness of the SFS group was significantly (*P* < 0.05) higher than that of the LSS group (Figure 2A) after 6 w of frozen storage, which differs from the results of Szymczak (4). In this study, after F–T cycles and frozen storage, a significant amount of water was released from the muscle of the SFS group, leading to a decrease in water content. Consequently, the hardness increased. With the increase of F–T cycles, the hardness of LSS group firstly increased and then decreased, which due to uneven distribution of water and salt before F–T cycles, and the decrease in hardness after multiple F–T cycles may be related to the decomposition of myofibrillar protein. The hardness of the LSS group showed no significant changes during frozen storage with F–T cycles. Salting treatment significantly (*P* < 0.05) enhanced the chewiness of muscle, since myogenic fibril protein structures are solubilized in the presence of salt. After 6 weeks of storage, the chewiness of the SFS group sample was significantly (*P* < 0.05) higher than that of the LSS group sample, due to a loss in moisture. The chewiness of the LSS group showed no significant changes during frozen storage with F–T cycles. This demonstrates salting could maintain the integrity of muscle, even after freezing. The springiness of the LSS group was higher than that of the SFS group. Moreover, it gradually increased with increasing F–T cycles but not significantly. Similar results were also reported by Jiang et al. (12) and might be related to the size of the ice crystals formed during freezing. The smaller ice crystals formed in the tissue after salting leads to less damage to the tissue structure; therefore, the tissue maintains better elasticity for recovery (21). The cohesiveness of the samples in the LSS group was significantly higher than that of the SFS group, and the SFS group showed a significant increase (*P* < 0.05) in cohesiveness after 6 weeks of storage, due to the positive effects of salting on the textural properties of meat, even after frozen storage (19).

### 3.3. Changes in TBARS and TVB-N

**FIGURE 3:**
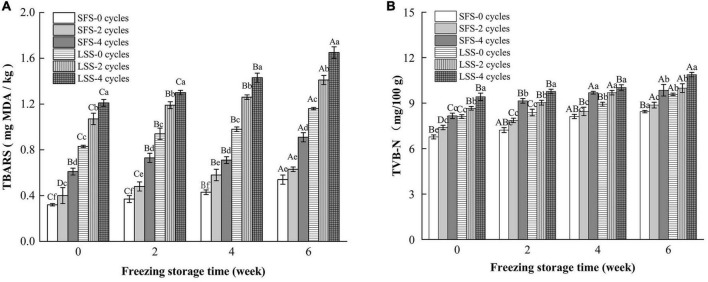
Changes in TBARS **(A)** and TVB-N **(B)** of LYC during frozen storage. SFS and LSS represent salt-free soaking group and low-salt soaking group, respectively. Lowercase letters represent significant differences between six samples during freezing, and capital letters represent significant differences between samples of different treatment groups during freezing (*P* < 0.05).

Lipid oxidation and protein deterioration are shown in Figure 3. Lipid oxidation is a critical biochemical reaction in meat products, and substances produced by lipid oxidation can be harmful, such as aldehydes (22). In this work, lipid oxidation in the LSS group was significantly higher than in the SFS group, and the number of F–T cycles and the storage period duration promoted lipid oxidation in both the LSS group and SFS group. Lipid oxidation is triggered by free radicals, and the pro-oxidative effect of NaCl was mainly due to its ability to disrupt the integrity of cell membranes, which transported salt into the lipid matrix (21). The redox balance in the fish muscle was disrupted, as it is ordinarily maintained by the endogenous antioxidant system in the living animal. Subsequently, a series of lipid oxidation chain reactions occurred during the salting process, where the redox balance was further displaced (23). Moreover, activated oxygen substances accumulated during subsequent repeated F–T cycles, leading to an acceleration of lipid oxidation (24).

Total volatile basic nitrogen is an important indicator of meat freshness and consists mainly of dimethylamine, trimethylamine, and ammonia substances, which are degradation products of proteins and nitrogenous compounds (25). Myofibrillar protein is a salt-soluble protein; therefore, salting could promote the oxidation of the protein. In Figure 3B, the TVB-N content in the LSS group was significantly higher than that in the SFS group, and there is a positive correlation between the number of F–T cycles and TVB-N accumulation. Even though the rates of TBARS and TVB-N production in the LSS group were faster than that in the SFS group, the meat was considered to be in the edible range after 6 w of frozen storage.

### 3.4. Color

**FIGURE 4:**
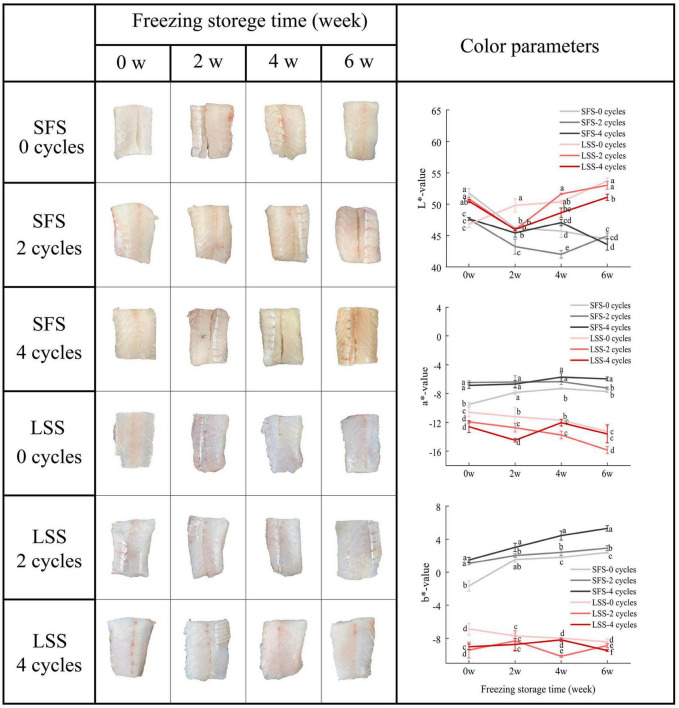
Changes in color of LYC during frozen storage. SFS and LSS represent the salt-free soaking group and low-salt soaking group, respectively. Different letters in the figure present significant differences between the six samples (*P* < 0.05).

The muscle status and color parameters of the LYC after F–T treatment and frozen storage are shown in Figure 4. Browning occurred in the SFS group with increasing F–T cycles and storage time. The LSS group samples had a vivid and smooth appearance during repeated F–T cycles and frozen storage, and the color of the muscle after four F–T cycles and 6 w of frozen storage showed a similar appearance to the fresh sample. In fact, the brightness was more desirable to consumers. In the present study, the L* value of the SFS group decreased with increasing F–T cycles after frozen storage. In contrast, the muscle of the LYC after low salting treatment maintained a bright appearance even after 4 F–T cycles and frozen storage. The L* value is considered to be associated with the water-holding capacity and microstructure, which affect the light-scattering properties of meat (26). During frozen storage, the a* value of the SFS group was significantly (*P* < 0.05) higher than that of the LSS group, which might be related to the decrease in myoglobin content caused by protein oxidation. Although the mechanism of post-salting meat discoloration is not clearly, this might related to the reduced antioxidant capacity of the tissue and the effect of salt on myoglobin stability (27). The b* values of the SFS group were significantly (*P* < 0.05) higher than that of the LSS group after frozen storage. White fish meat is prone to browning if exposed to temperature fluctuations during storage, and repeated F–T cycles significantly (*P* < 0.05) increased the b* value of the SFS group. The biochemical reactions in the tissues are related to salting and frozen storage, in particular lipid and protein oxidation. Freezing effectively inhibits microbial growth and enzymatic activity to extend the shelf life, but the unfrozen portion of the tissue still undergoes physicochemical reactions.

### 3.5. Moisture

**FIGURE 5:**
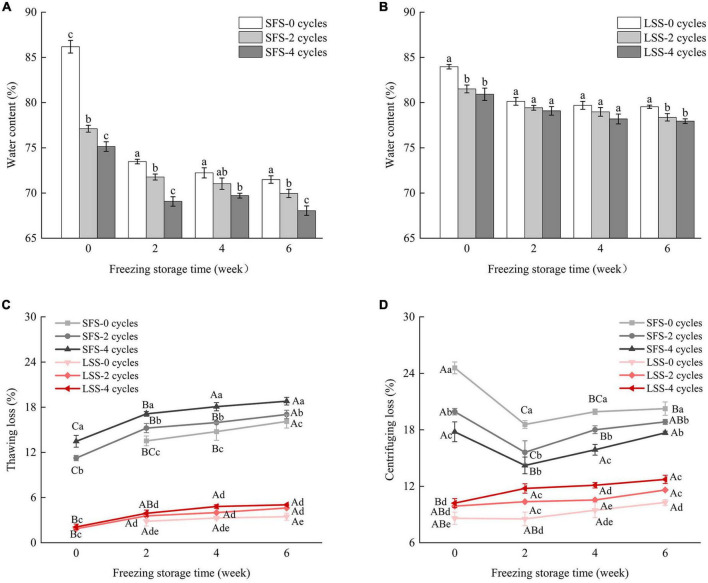
Changes in moisture content of LYC during frozen storage. **(A)** Water content of SFS group; **(B)** water content of LSS group; **(C)** thawing loss; **(D)** centrifuging loss. SFS and LSS represent the salt-free soaking group and low-salt soaking group, respectively. Lowercase letters represent significant differences between six samples during freezing, and capital letters present significant differences between samples of different treatment groups during freezing (*P* < 0.05).

The changes in the water content of different samples are shown in Figure 5. Water holding capacity is the ability of a food product to retain its original and additional moisture. The SFS group samples acquired moisture after salting, and the water content decreased significantly after F–T cycles and frozen storage (Figure 5A). It is possible that the extracted water formed large ice crystals after freezing, and the growth of ice crystals after repeated F–T cycles and frozen storage caused irreversible mechanical damage to the muscle. However, a significant (*P* < 0.05) decrease was observed in the water content of the fish, due to an increase in the thawing loss rate (28). There was no significant (*P* < 0.05) difference in water content between the LSS group after two F–T cycles and four F–T cycles. The water content of the LSS group was higher than that of the SFS group after frozen storage, as shown in Figures 5A, B, indicating salting could protect tissue and cellular structure from damage and significantly (*P* < 0.05) increase the water-holding capacity of muscle (13). Thawing loss was the main factor contributing to the reduced yield, with the SFS group showing a significantly higher thawing loss than the LSS group (Figure 5C). This might be the result of salt enhancing the binding between water and myofibrillar protein, especially myofibrillar protein swelling after extracting water (29). The high centrifuging loss of the SFS group was observed during the initial storage period, due to the relatively low rate of thawing and the decrease in water content, simultaneously (Figure 5D). The increase in centrifuging loss after 2 weeks of frozen storage was due to the irreversible damage to the muscle fibers caused by the accumulation and enlargement of ice crystals during frozen storage (30). Centrifuging losses in the LSS group showed slight changes during frozen storage, even after four F–T cycles.

### 3.6. Observation of tissue microstructure

**FIGURE 6:**
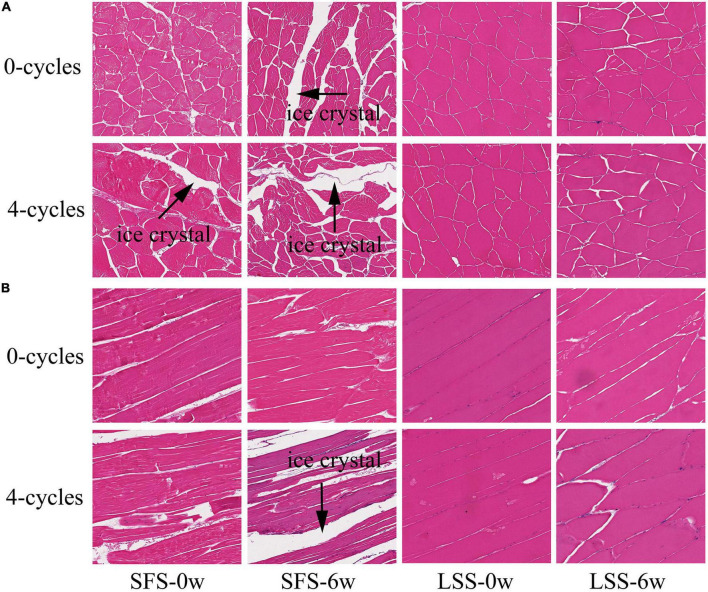
Structural observation of LYC. SFS and LSS represent the salt-free soaking group and low-salt soaking group, respectively. Panels **(A,B)** showed the cross-section and vertical-section, respectively. The magnification of images is 10 times.

The changes in the microstructure of the LYC after F–T cycles and frozen storage are shown in Figure 6. As observed in the longitudinal section, which hindered the recovery of the tissue (Figure 6A) (10). Myogenic fibers of muscle in the LSS group before storage and F–T treatment became plump and regular after taking on water and salt, and the reduction or disappearance of intracellular cavities helped the tissue to increase the elasticity and water retention capacity, even after four T-F cycles (21). Myogenic fibers with inconspicuous boundaries and amorphous structures were observed in the SFS group after 6 weeks of frozen storage. The microstructure further deteriorated, especially after repeated F–T cycles, due to the enormous pressure of ice crystals. The crystals twisted and broke myofibrils and released cellular material into the intercellular spaces. Expanded extracellular spaces might form channels, consequently affecting the quality of the meat and causing a loss of nutrients after frozen storage (5). The muscle fiber boundaries were apparent after 6 weeks of frozen storage in the LSS group, and this might be related to the loss of some water after frozen storage. They remained clear after four F–T cycles, although the boundaries of muscle bundles became less distinct (black pointed tip indicates). This was possibly related to the degradation of coarse tissue caused by salting. The morphology of the ice crystals is an essential factor in determining the quality of the fish, particularly in terms of water-holding capacity and texture. In general, tiny ice crystals are favorably associated with a reduction in thawing loss and damage to the structure of myogenic fibrous proteins. The present study confirmed salting could restore the histomorphology of meat after repeated F–T cycles and counteract the intracellular damage (29). The change in tissue structure in the LSS group might be due to the swelling of the myofilament lattice and the solubilization of myogenic fibrin, which could enhance the water-holding capacity of the muscle.

### 3.7. SDS-PAGE patterns of protein

**FIGURE 7:**
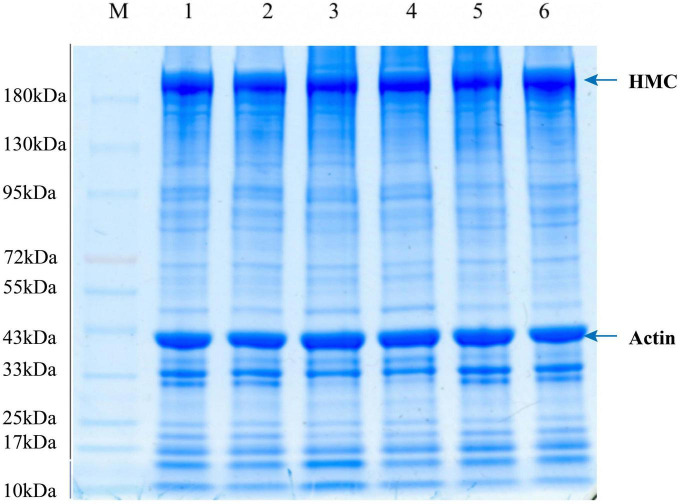
Changes in SDS-PAGE of LYC during frozen storage. 1, 2 are fresh samples of LYC, 3, 4, and 5, 6 are SDS-PAGE profiles of total protein of LSS group and SFS group after 4 F-T cycles and 6w frozen storage, respectively.

The SDS-PAGE results of LYC samples treated with a low salt solution are shown in Figure 7. Bands around 220 kDa were found in lanes 1, 2, 3, 4, 5, and 6, which were attributed to the heavy chains of myosin (31). Protein degradation, fragmentation, and aggregation during repeated F–T cycles and frozen storage might be caused by the high activity of histone proteases (32). A reduction in 97 kDa protein was found in the SFS group samples, compared to LSS group samples. A water-soluble protein with a molecular weight of 97 kDa, tentatively designated as glycogen phosphorylase, was observed in the SFS group (12). This result suggested that freezing may affect meat quality by influencing the enzymes involved in biochemical muscle reactions. In addition. Peptides at 32 kDa decreased, and the peptides at 48 and 64 kDa increased in the LSS group after frozen storage. Which suggests protein exhibited aggregation due to salting. Thus, changes in protein structure occurred during frozen storage in the LSS group and SFS group samples. The molecular weight protein segments around of 45 kDa were tentatively identified as myosin (12). The instability of actin and myogenic fibrils in meat might be attributed to different protein concentrations and salt condensation effects during freezing. Salting could alter the extraction and/or solubilization of myofibrillar proteins, as well as influence meat product quality and storage stability. Therefore, the quality and storage stability of the final product after further processing might be related to the protein characteristics after salting.

## 4. Conclusion

In this work, the effects of low salting on the quality changes to LYC meat during repeated F–T cycles and frozen storage were investigated. The microstructure of the LSS group was more stable than that of the SFS group. In addition, the water-holding capacity of the LSS group was significantly improved. The results provided a theoretical basis and applicable reference for the optimization of the transportation, processing, and storage conditions of aquatic products. A limitation of the salting treatment was its tendency to promote lipid and protein oxidation during repeated F–T cycles. Therefore, future research should focus on the lipid and protein oxidation of meat products during transportation after salting.

## Data availability statement

The raw data supporting the conclusions of this article will be made available by the authors, without undue reservation.

## Ethics statement

Ethical review and approval was not required for the animal study because disintegrate according to the norms.

## Author contributions

HB and JZ contributed to the investigation, methodology, formal analysis, data curation procedures, and writing the original manuscript draft. ML, YC, JY, and CM contributed to the investigation and data curation procedures. YG contributed to conceptualization, project administration, supervision, and reviewing and editing the draft. SD contributed to validation and reviewing and editing the draft. All authors have read and agreed to the published version of the manuscript.

## References

[1] FAO. *Fisheries and Aquaculture Division [Online].* Rome: Food and Agriculture Organization of the United Nations (2022).

[2] Soottawat B Seymour TA Morrissey MT Haejung A. Physicochemical Changes in Pacific Whiting Muscle Proteins. *J Food Sci.* (1997) 62:729–33.

[3] Ando Y Hagiwara S Nabetani H Okunishi T Okadome H. Impact of ice crystal development on electrical impedance characteristics and mechanical property of green asparagus stems. *J Food Eng.* (2019) 256:46–52. 10.1016/j.jfoodeng.2019.03.019

[4] Szymczak M Kamiński P Felisiak K Szymczak B Dmytrów I Sawicki T. Effect of constant and fluctuating temperatures during frozen storage on quality of marinated fillets from atlantic and baltic herrings (*Clupea harengus*). *LWT.* (2020) 133:109961. 10.1016/j.lwt.2020.109961

[5] Lan W Hu X Sun X Zhang X Xie J. Effect of the number of freeze-thaw cycles number on the quality of pacific white shrimp (*Litopenaeus vannamei*): An emphasis on moisture migration and microstructure by Lf-Nmr and Sem. *Aquac Fish.* (2020) 5:193–200. 10.1016/j.aaf.2019.05.007

[6] Dalvi-Isfahan M Jha P Tavakoli J Daraei-Garmakhany A Xanthakis E Le-Bail A. Review on identification, underlying mechanisms and evaluation of freezing damage. *J Food Eng.* (2019) 255:50–60. 10.1016/j.jfoodeng.2019.03.011

[7] Du X Chang P Tian J Kong B Sun F Xia X. Effect of ice structuring protein on the quality, thermal stability and oxidation of mirror carp (*Cyprinus Carpio* L.) induced by freeze-thaw cycles. *LWT.* (2020) 124:109140. 10.1016/j.lwt.2020.109140

[8] Szymczak M. Effect of technological factors on the activity and losses of Cathepsins B, D and L during the marinating of atlantic and baltic herrings. *J Sci Food Agric.* (2017) 97:1488–96. 10.1002/jsfa.7889 27391990

[9] Geleijnse J Witteman J Stijnen T Kloos M Hofman A Grobbee D. Sodium and potassium intake and risk of cardiovascular events and all-cause mortality: The rotterdam study. *Eur J Epidemiol.* (2007) 22:763–70. 10.1007/s10654-007-9186-2 17902026 PMC2071962

[10] Zhang B Cao H Lin H Deng S Wu H. Insights into ice-growth inhibition by trehalose and alginate oligosaccharides in peeled pacific white shrimp (*Litopenaeus vannamei*) during frozen storage. *Food Chem.* (2019) 278:482–90. 10.1016/j.foodchem.2018.11.087 30583401

[11] Yu J Lu K Zi J Yang X Xie W. Characterization of aroma profiles and aroma-active compounds in high-salt and low-salt shrimp paste by molecular sensory science. *Food Biosci.* (2022) 45:101470. 10.1016/j.fbio.2021.101470

[12] Jiang Q Jia R Nakazawa N Hu Y Osako K Okazaki E. Changes in protein properties and tissue histology of tuna meat as affected by salting and subsequent freezing. *Food Chem.* (2019) 271:550–60. 10.1016/j.foodchem.2018.07.219 30236715

[13] Jiang Q Nakazawa N Hu Y Osako K Okazaki E. microstructural modification and its effect on the quality attributes of frozen-thawed bigeye tuna (*Thunnus Obesus*) meat during salting. *LWT.* (2019) 100:213–9. 10.1016/j.lwt.2018.10.070

[14] AOAC. *Official Methods of Analysis. Washington, Dc: Association of Official Analytical Chemists Inc.* Washington, DC: AOAC international (1999).

[15] Jiranuntakul W Nakwiang N Berends P Kasemsuwan T Saetung T Devahastin S. Physicochemical, microstructural, and microbiological properties of skipjack tuna (*Katsuwonus Pelamis*) after high-pressure processing. *J Food Sci.* (2018) 83:2324–36. 10.1111/1750-3841.14318 30106476

[16] G5009.181. *Determination of Malondialdehyde in Food.* Beijing: National Food Safety Standard of China (2016).

[17] G5509.228. *Determination of Volatile Salt-Based Nitrogen in Food.* Beijing: National Food Safety Standard of China (2016).

[18] Cao H Zhu H Wang Q Fan D Huang J Zhao J Intervention on activity and structure of Cathepsin L during surimi gel degradation under microwave irradiation. *Food Hydrocoll.* (2020) 103:105705. 10.1016/j.foodhyd.2020.105705

[19] Jiang Q Nakazawa N Hu Y Wang X Osako K Okazaki E. Evolution of tissue microstructure, protein properties, and oxidative stability of salted bigeye tuna (*Thunnus Obesus*) meat during frozen storage. *LWT.* (2021) 149:111848. 10.1016/j.lwt.2021.111848

[20] Deng J. Effect of freezing and forzen storage on salt penetratin into fish muscle immered in brine. *Food Sci.* (1977) 42:348–51.

[21] Jiang Q Nakazawa N Hu Y Osako K Okazaki E. Changes in quality properties and tissue histology of lightly salted tuna meat subjected to multiple freeze-thaw cycles. *Food Chem.* (2019) 293:178–86. 10.1016/j.foodchem.2019.04.091 31151599

[22] Huang L Xiong Y Kong B Huang X Li J. Influence of Storage temperature and duration on lipid and protein oxidation and flavour changes in frozen pork dumpling filler. *Meat Sci.* (2013) 95:295–301. 10.1016/j.meatsci.2013.04.034 23747621

[23] Mariutti L Bragagnolo N. Influence of salt on lipid oxidation in meat and seafood products: A review. *Food Res Int.* (2017) 94:90–100. 10.1016/j.foodres.2017.02.003 28290372

[24] Yu Y Yang S Lin T Qian Y Xie J Hu C. Effect of cold chain logistic interruptions on lipid oxidation and volatile organic compounds of salmon (*Salmo salar*) and their correlations with water dynamics. *Front Nutr.* (2020) 7:155. 10.3389/fnut.2020.00155 33015126 PMC7509473

[25] Fan H Luo Y Yin X Bao Y Feng L. Biogenic amine and quality changes in lightly salt- and sugar-salted black carp (*Mylopharyngodon piceus*) Fillets Stored at 4 Degrees C. *Food Chem.* (2014) 159:20–8. 10.1016/j.foodchem.2014.02.158 24767022

[26] Hughes J Oiseth S Purslow P Warner R. A structural approach to understanding the interactions between colour, water-holding capacity and tenderness. *Meat Sci.* (2014) 98:520–32. 10.1016/j.meatsci.2014.05.022 25034451

[27] Jiang Q Du Y Nakazawa N Hu Y Shi W Wang X Effects of frozen storage temperature on the quality and oxidative stability of *Bigeye tuna* flesh after light salting. *Ing J Food Sci Tech.* (2022) 57:3069–77. 10.1111/ijfs.15636

[28] Fan L Ruan D Shen J Hu Z Liu C Chen X The role of water and oil migration in juiciness loss of stuffed fish ball with the fillings of pig fat/meat as affected by freeze-thaw cycles and cooking process. *LWT.* (2022) 159:113244. 10.1016/j.lwt.2022.113244

[29] Offer G Trinick J. On the mechanism of water holding in meat: The swelling and shrinking of myofibrils. *Meat Sci.* (1983) 8:245–81. 10.1016/0309-1740(83)90013-X 22055626

[30] Vieira C Diaz M Martinez B Garcia-Cachan M. Effect of frozen storage conditions (Temperature and Length of Storage) on microbiological and sensory quality of rustic crossbred beef at different states of ageing. *Meat Sci.* (2009) 83:398–404. 10.1016/j.meatsci.2009.06.013 20416701

[31] Lopez-Pedrouso M Perez-Santaescolastica C Franco D Fulladosa E Carballo J Zapata C Comparative proteomic profiling of myofibrillar proteins in dry-cured ham with different proteolysis indices and adhesiveness. *Food Chem.* (2018) 244:238–45. 10.1016/j.foodchem.2017.10.068 29120776

[32] Cai L Nian L Cao A Zhang Y Li X. Effect of carboxymethyl chitosan magnetic nanoparticles plus herring antifreeze protein on conformation and oxidation of myofibrillar protein from red sea bream (*Pagrosomus major*) after freeze-thaw treatment. *FABT.* (2019) 13:355–66. 10.1007/s11947-019-02384-x

