# Supplementary figures and images for: Effect of freezing-thawing on the quality changes of large yellow croaker treated by low-salt soaking during frozen storage
Source: Front Nutr. 2023 Jan 10;9:1103838. doi: 10.3389/fnut.2022.1103838 (PMC9872034; doi:10.3389/fnut.2022.1103838)

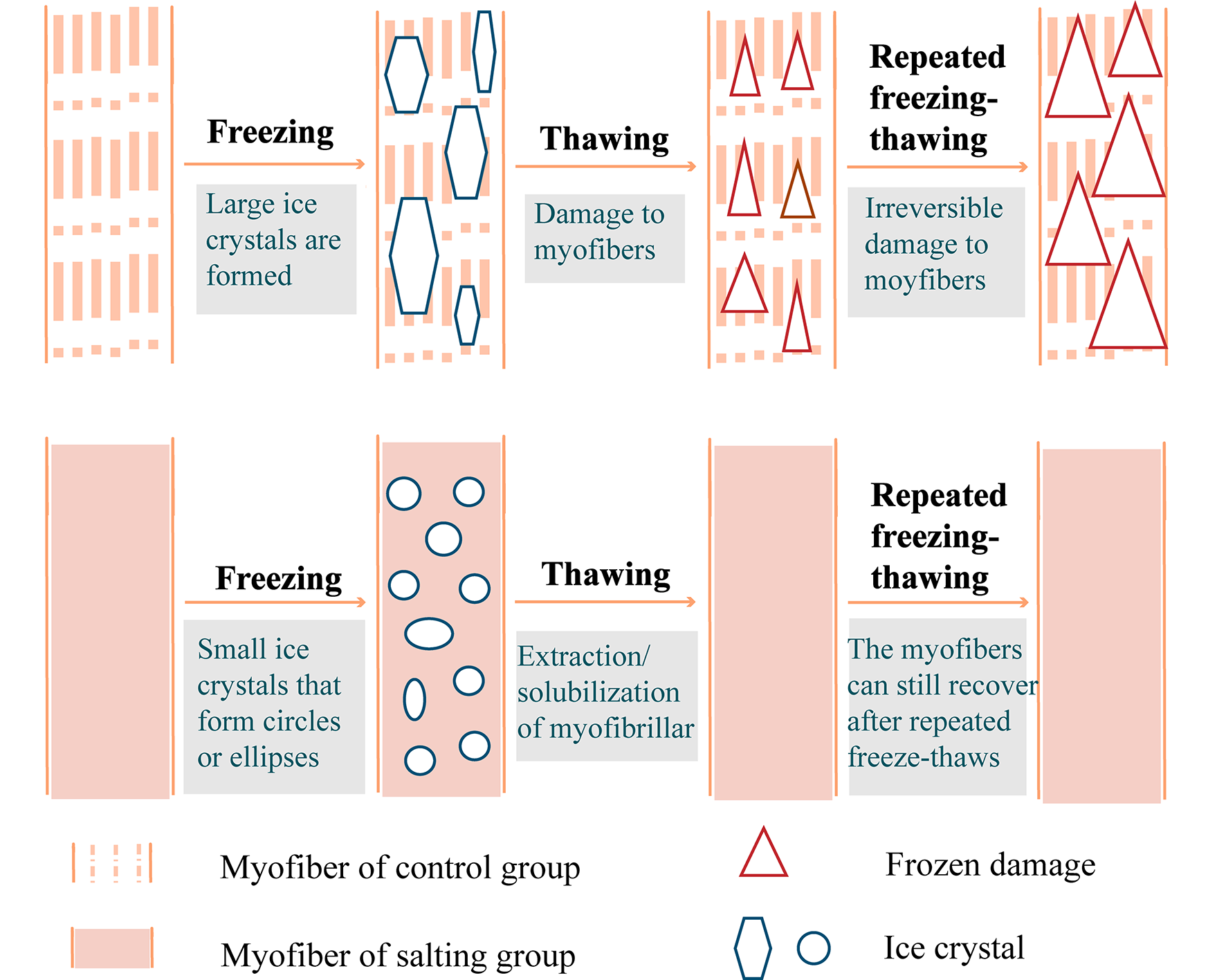

Supplement: Supplementary file 1 [file Image_1.TIF]
